# Patient and public involvement and engagement (PPIE): how valuable and how hard? An evaluation of ALL_EARS@UoS PPIE group, 18 months on

**DOI:** 10.1186/s40900-024-00567-1

**Published:** 2024-04-11

**Authors:** Kate Hough, Mary Grasmeder, Heather Parsons, William B Jones, Sarah Smith, Chris Satchwell, Ian Hobday, Sarah Taylor, Tracey Newman

**Affiliations:** 1 Clinical and Experimental Sciences, Faculty of Medicine, University of Southampton, Southampton, UK; 2 Auditory Implant Service, University of Southampton, Southampton, UK; 3 NIHR Research Design Service, University of Southampton, Southampton, UK; 4 Southampton Centre for Research Involvement and Engagement, University Hospital Southampton, Southampton, UK; 5 Wessex Public Involvement Network, University Hospital Southampton NHS Foundation Trust, Southampton, UK; 6 ALL_EARS@UoS PPIE Group Member, University of Southampton, Southampton, UK

**Keywords:** Patient and public involvement, Evaluation, Hearing loss, Audiology, Community engagement, Standards

## Abstract

**Background:**

ALL_EARS@UoS is a patient and public involvement and engagement (PPIE) group for people with lived experience of hearing loss. The purpose of the group is to share experiences of hearing loss and hearing healthcare, inform research and improve services for patients at University of Southampton Auditory Implant Service. A year after inception, we wanted to critically reflect on the value and challenges of the group. Four members of ALL_EARS@UoS were recruited to an evaluation steering group. This paper reports the evaluation of the group using the UK Standards for Public Involvement.

**Methods:**

An anonymous, mixed-methods questionnaire was co-designed and shared with members of ALL_EARS@UoS using an online platform. The questionnaire was designed to capture satisfaction, individual feedback through free-text answers, and demographic information. Descriptive statistics have been used to express the satisfaction and demographic data. Reflexive thematic analysis has been used to analyse the free-text responses. Group engagement and activity data over time were monitored and collected.

**Results:**

The questionnaire response rate was 61% (11/18). Areas identified as strengths were ‘Communication’ and ‘Working together’. Five themes were developed from the thematic analysis; (1) Increased knowledge and awareness around the topic of hearing health for group members and wider society, (2) supporting research, (3) inclusivity within the group, (4) opportunity to make a difference for people in the future and (5) running of the group/group organisation. The data highlighted the value and challenges of PPIE. Members described feeling listened to and appreciation of being able to share experiences. Time of day and meeting format were identified as challenges as they affected who could attend the meetings. The ability to secure and maintain sufficient funding and time to support inclusive and diverse PPIE activities is a challenge for researchers.

**Conclusions:**

We have identified how PPIE added value to both group members and researchers, emphasising the true benefit of PPIE. We have highlighted challenges we are facing and our plan to tackle these. We aim to continue to develop and sustain a group that reflects the diversity of the Deaf/deaf or hard of hearing community and of our local community.

**Supplementary Information:**

The online version contains supplementary material available at 10.1186/s40900-024-00567-1.

## Abstract (key points)

Patient and public involvement and engagement enables members of the public to influence what research happens. We have established a group called ALL_EARS@UoS for people with lived experience of hearing loss. This is so we can work together and learn from our group members’ experiences, to inform our research into hearing loss. The members of ALL_EARS have helped to decide the aims, objectives, and principles of the group. This paper is a report of the value and challenges of the first year of the group. We recruited members of the group to work with us to design a survey. This paper describes the findings from the survey. Our members described benefits, ranging from supporting research to being able to share their experience with others. A challenge for members is the difficulty in being involved alongside their other commitments. A challenge for researchers is the funding and time needed to maintain the group. Other challenges include ensuring the group is diverse and that we understand and meet the needs of our members. We report on how we will address these challenges, so we can continue to work in partnership as a group.

**Supplementary Information:**

The online version contains supplementary material available at 10.1186/s40900-024-00567-1.

## Background

Patient and public involvement (PPI) refers to research being carried out ‘with’ or ‘by’ members of the public rather than ‘to’, ‘about’ or ‘for’ them [1]. It is designed to ensure that lived experience influences the design and delivery of research so that it translates into benefit for people. The fundamental principle, which led to public involvement in research, is that people who are affected by the outcomes or process of research have the right to have a say in what, and how, research is undertaken [2]. The benefits of PPI across health and social care research are being increasingly recognised [2–4] and there is a growing emphasis on involving the public and patients across all stages of the research process. Leading UK research funding bodies such as National Institute of Health and Care Research (NIHR) and UK Research and Innovation (UKRI), expect involvement of members of the public in all stages of the research that they fund. Public engagement differs from patient and public involvement (PPI). The National Co-ordinating Centre for Public Engagement (NCCPE) defines public engagement as ‘the myriad of ways in which the activity and benefits of higher education can be shared with the public. Engagement is by definition a two-way process, involving interacting and listening, with a goal of generating mutual benefit’ [5]. Throughout this paper, we refer to our activity as PPIE to encompass patient and public involvement *and* engagement. Within the paper, other literature may use the term PPI or PPIE and so when referring to a specific aspect of our work, we will describe it in full as engagement or involvement.

Approaches to patient and public involvement include consultation, collaboration, co-production, and patient led/user-controlled research [1]. These involve differing levels of commitment and responsibility and sharing of power. Table 1 highlights the differences between involvement, engagement, and participation in research with examples for each.

**Table 1:** Types of patient and public involvement and engagement and participation in research

Involvement		Examples
Consultation	Asking members for their views and using these views to inform decision making.	- Discussing research project ideas with patients and the public to find out how relevant they think the projects are. - Asking patient and carers to read, and provide feedback on, documents in the research design pathway.
Collaboration/co-production	Where decisions about the research are shared between researchers and members of the public.	- PPI members collaborating with researchers to develop a grant application. - PPI members being involved with interviews with research participants for qualitative data collection.
Patient-led	Where research is controlled, directed, and managed by service users and their service user organisations.	- PPI members as researchers on a project where they design, initiate, and deliver the research.
Engagement	Where information and knowledge about research is provided and disseminated through a range of activities.	- Running a stall/exhibit at a science festival. - Giving a public lecture or talk to inform the local community.
Participation	Where members of the public take part in the research study.	- Taking part in a clinical trial. - Completing a questionnaire for a research study.

The benefits of incorporating PPIE in health research are many and can be valuable for members of the public, patients, researchers, community groups and clinicians [6, 7]. There is recognition that involvement can improve the quality and relevance of research to patients and the public [2, 8]. Ethical and democratic benefits include the right of the public to have a voice in how public money is being spent and what research the money is funding. When effective, this results in greater transparency and accountability to funders and the public. Patient and public contribution to research proposal development and study design can increase the chance of funding due to the early input from contributors, increasing the clarity, credibility, and relevance of the study [9, 10]. An ethical argument in favour of PPI is that the individual has the right to be fully involved with any health care or research intervention being done ‘to’ them as a person [11].

For public involvement to be effective in improving research quality and relevance, and improve health outcomes, the patient and public members/contributors must reflect the diversity of our communities. Recruitment of a diverse and inclusive group of people is a recognised challenge for PPI [12]. The NIHR public contributors’ feedback survey carried out between December 2018 and January 2019, highlighted that the NIHR public involvement community [13] did not reflect the population diversity of England and Wales. Young people and minority ethnic communities were typically under-represented. Younger people made up only 2% (under 25 years old) and 14% (age 26–49 years) of surveyed public contributors. Only 2% of surveyed public contributors came from Asian ethnic groups and only 3% represented black ethnic groups [13]. According to 2021 Census, 81.7% of the population in England and Wales are white. 9.3% identify themselves as Asian, 4% as Black, 2% as mixed and 2.1% as other ethnic group [14].

The term *‘underserved communities’* refers to the people who the research community need to do more with to provide a better service for [15]. Characteristics of people in underserved groups include lower inclusion and involvement in research compared to other groups, differences in how the group accesses, engages with, and responds to, existing healthcare interventions compared to other groups and high healthcare burden that is not matched by the volume of research carried out for the group [15]. Some examples include minority ethnic groups, socioeconomically disadvantaged groups, people in alternative residential circumstances (e.g. migrants, the homeless and prisoners), people with a physical or learning disability and carers. Underserved communities often face barriers in accessing healthcare information and services, as well as getting involved in research. Barriers to inclusion and involvement include physical disability, lack of trust and interest in trials and health research, cultural barriers, and specific health fears. It is our responsibility as researchers to work to remove the barriers by making a conscious effort to go into communities, to raise awareness of research and to invite people to become participants.

Setting out to work with people with hearing loss/ profound hearing loss adds challenges if meetings are to be inclusive. This very challenge to make them inclusive highlights the barriers that people with hearing loss are likely to encounter when their communication requirements are not considered. Meetings need to be designed to support participation through being accessible for all members, irrespective of their hearing status, to engage and contribute. There is diversity within deafness. The needs and communication methods vary between people who identify as deaf (who have severe to profound hearing loss), Deaf (who identify as part of the Deaf community), and hard of hearing (who have hearing loss – often mild to moderate). Several communication methods and tools are used by d/Deaf and hard of hearing people including lip-reading, British Sign Language (BSL), Sign Supported English and the use of assistive technologies such as hearing aids and cochlear implants. D/deafness can co-exist with other sensory disabilities, including poor or little sight. Adjustments need to support participation in meetings and other activities. Language service professionals including sign language interpreters, captioning/note takers and the use of deafblind manual can help bridge what is otherwise a communication gap.

There is some published evidence of the involvement of patients and members of the public in audiology research [16, 17]. This shows the nature and context of the involvement and how the PPI was conducted, varies. One case describes the development of a PPIE group to support an already established research portfolio at an NHS Audiology and ENT department and describes the benefits of the group to specific projects [16]. Another study describes examples of how members of the public have been involved in different hearing research projects at a UK research and teaching centre for Audiology and Deafness and the opportunities and benefits of each [17]. In each of these cases PPI contributors were recruited for each project, a common occurrence in PPI, rather than building a group of members who could potentially contribute to, and influence, the growth and direction of a body of research over time.

Our goal was to create an active and enduring PPIE group of patients and members of the public to input to both clinical activities at the University of Southampton Auditory Implant Service (USAIS) and research across the University of Southampton. We aimed to create a partnership which empowers the members of the group to contribute to, and influence, the research being carried out to benefit people with hearing loss and their families. We aspired to establish and sustain a group where there is mutual reward, respect, and benefit for all members of the group. The main drivers of this work were to increase public awareness of hearing loss and hearing healthcare; the use and value of assistive technologies such as cochlear implants; improve access to cochlear implants and to improve hearing outcomes for people with hearing loss and/or a cochlear implant.

An approach to the NIHR Research Design Service in November 2021 to establish whether funding existed to establish a hearing loss PPIE group, led to support from PPI officers within NIHR that started the development our PPIE strategy. We found there was no PPIE specific funding available at that time. This highlighted the importance of building costed applications for research bids to support a hearing loss PPIE group. Funding at the early stages of PPIE is essential to support the time needed to develop and grow relationships, to manage the administration of a group and to reach out into the community. The notion of a group was first advertised to patients at the University of Southampton Auditory Implant Service (USAIS) in March 2022 through Twitter (X), the USAIS website, and flyers in the clinic reception area. Our first meeting, with eight group members, was held in May 2022. Between May and December several more meetings were held, with a steady increase in number of members in the group. By December 2022, it was clear that there was value to the group members and that we needed to expand and broaden the membership. We advertised through local support groups, social media and at local community groups funded through a small community-engagement project. Between 12 and 14 members participated in meetings in January, May, and July 2023. By July, ten of the group members were people who had joined after discovering the group through our community engagement and outreach activities.

As the group developed and evolved, we worked with members of the group to co-design and establish the elements of the group. These included the group name [ALL_EARS@UoS], the logo, ethos, aims and objectives all of which was written up and agreed in a terms of reference document. Our aims for ALL_EARS@UoS are to work with the group members to identify and prioritise the most relevant research. We aim to involve members at all stages of the research process – from as early as possible in this process. To support this, we held a training and information session about the complexities of the research process and highlighted all the points where members could get involved. By taking the time to learn about our member’s experiences, we started to be able to identify the issues that are most important for people living with hearing loss. We want to turn these issues into research questions then turn these into research projects.

We plan to sustain the group by ensuring that researchers and health care professionals are aware of the group, and that they approach us early in their research planning. We have established a framework to describe how researchers can work with members of our group, share ideas and receive feedback from members of the group about project ideas (see additional file 4). We ask that researchers who work with the group build appropriate levels of costings for PPIE into research applications, consider giving some of their time to support PPIE and to think about how they can support the group to be recognised more widely.

With the growing need and expectation for PPIE to be embedded within research comes a growing need to evaluate, reflect and report upon current practice. The frameworks, tools, and methods for evaluating patient and public involvement are growing rapidly. The international literature evaluating the impact of PPI has tripled in the last 10 years [18]. A scoping review of methods to measure and evaluate citizen engagement (i.e. partnering with members of the public) in health research identified a variety of approaches including frameworks, discussion-based, survey-based and other methods [19]. These methods commonly gather the perceptions of those involved, and are often focused on empowerment, impact, respect, support, and value [19]. A recent systematic review identified 65 different frameworks designed to map, evaluate, or report on PPIE [20]. These frameworks fit into five main categories; power-focused, priority setting, study focused, report-focused, partnership focused. The evaluation of PPIE is essential to inform and enhance future practice.

Throughout this manuscript, we will report on and discuss the context, outcomes and impacts of our PPIE group based on data we have collected through monitoring over time (e.g. number of meetings held, number of contributions to research projects) alongside data collected from an anonymous evaluation questionnaire that was shared with group members.

## Aims

To evaluate (1) the engagement, outputs, and outcomes of the PPIE group by monitoring activities over time (2) the impact, effectiveness, and engagement of the PPIE group ALL_EARS@UoS using an anonymous questionnaire. Our objective was to gather data to report on the development, progression, and effectiveness of ALL_EARS PPIE group.

## Methods

### Establishing the PPIE group

The people who have joined ALL_EARS@UoS have lived experience of hearing loss and/or cochlear implants. This could mean personal experience of deafness/hearing loss. Equally it could mean a partner, family member, parent, or carer of someone with deafness/hearing loss or someone who has a cochlear implant/s. There is diversity in the severity of hearing loss/deafness between group members, this is exemplified by some people wearing a hearing aid/s, some have a cochlear implant/s, some wear both a hearing aid and cochlear implant and some people wear neither but may have a hearing dog.

#### Co-development of the group

After the group was first advertised we had a small group of interested members who attended our first meeting in May 2022. We worked with the group and members who joined over the following months, to co-develop and build the group. Group facilitation supported the discussion and establishment of the aims and objectives of the group at a meeting in September 2022. Through group working we co-edited a paragraph written by a member of the group which outlined the group and the group’s aspirations. This text is displayed on the front page of the group website [21]. Several group members responded to a request to suggest names for the group. These were collected and an anonymous poll was used to enable all members of the group to vote to select the preferred the name. A similar process was used to generate a logo for the group. Drawings produced by group members were turned into electronic graphic images and a poll was run to identify the most popular image.

PPI was used in the development of the group’s aims, objective, ethos, and roles/responsibilities, which were established and agreed upon as the group grew and developed. Currently, the nature of involvement with our group is a combination of consultation and collaboration. Our goal is to reach an optimal level of involvement which is co-production of research.

### Monitoring PPIE activity

Since establishing the group we have monitored or collected data to capture the group’s activities, meetings, and outputs over time. This included the number of members, where they were recruited from, the number of meetings and attendees at each meeting, the cost of each meeting (refreshments, note taker, involvement fees and expenses), the projects and grants the group has been involved with, the public engagement activities and events and the type and number of outputs (e.g. newsletter, blog).

### Evaluation of the PPIE group using an anonymous questionnaire

#### Planning and questionnaire design

For the formal evaluation, ALL_EARS members were invited to join the evaluation steering group. Four group members agreed to join (two members subsequently left the group at data analysis stage due to increased family commitments).

Steering group members were involved in the design and development stages of the evaluation by suggesting questions to include and topics to cover and through multiple iterations of the draft questionnaire. Using the feedback, alongside the UK Standards for Public Involvement [22], a full draft of the questionnaire was produced and circulated to the steering group.

There are six distinct standards identified in the UK Standards for Public Involvement. These include communication, governance, impact, working together, support and learning, and inclusive opportunities [22], as outlined in Table 2. These standards provide a benchmark for researchers to work towards to ensure effective patient and public involvement and standards were co-produced by researchers, funders, public partners, and involvement practitioners [23]. The UK Standards for Public Involvement have been used to design and structure a process for reflection on, and evaluation of, PPI [24–26]. To evaluate our progress against the standards, we used a series of statements which aligned to the standards to generate the questionnaire. For example, for Communication – the following statements included:

- *There are clear and informative communications about upcoming meetings, written work, and activities.*
- *There are regular opportunities to offer feedback about meetings, project ideas and activities.*
- *My feedback is gathered, acted on and shared back to the group.*

The group members were asked to use a rating scale to identify how much they agreed with the statements from Strongly agree, agree, neither agree nor disagree, disagree, and strongly disagree.

**Table 2:** UK Standards for public involvement [22]

UK Standards for Public Involvement	
Communications	Use plain language for well-timed and relevant communications, as part of involvement plans and activities.
Governance	Involve the public in research management, regulation, leadership, and decision making.
Impact	Seek improvement by identifying and sharing the difference that public involvement makes to research.
Working together	Work together in a way that values all contributors and build and sustains mutually respectful and productive relationships.
Support and learning	Offer and promote support and learning opportunities that build confidence and skills for public involvement in research.
Inclusive opportunities	Offer public involvement opportunities that are accessible and that reach people and groups according to research needs.

#### Questionnaire structure

The questionnaire was a combination of questions that asked participants to produce a rating; complete open-text answers and report demographic data using a check box list. This included age, gender, and ethnicity, and was captured to determine how the population of our group reflects the population of patients at USAIS and of the population within the catchment area for the service (The South of England). Collecting this data early in the lifetime of the group enables us to consider whether and how we might improve diversity within the group.

As this PPIE group is still relatively young, we opted to include questions that we would like to ask in the future around Governance and asked respondents to share their thoughts on this area. The four questions were: (1) Public involvement plans are in place and these plans are regularly monitored, reviewed, and reported on. (2) Throughout the organisation, there is visible and accountable responsibility for public involvement. (3) Realistic resources such as money, staff, time are allocated for public involvement. (4) The privacy of personal information is protected by collecting and using it in a suitable way.

Four open-ended questions were included: (1) What worked well? (2) What would you like to see improved? (3) What do you see as the impact of being involved within this group? (4) Describe any benefits of being part of ALL_EARS@UoS.

The questionnaire contained 36 questions split across ten sections. The estimated time to complete the questionnaire was 13 min. Immersive Reader, which has various tools to increase accessibility e.g. changing font size and text-to-speech, was available. See additional file 2 for a copy of the questionnaire.

#### Ethics

Ethical approval for the questionnaire was granted by the University of Southampton Ethics Committee, reference number ERGO/FEPS/ 81,056.

#### Consent

Participants were asked to consent to taking part in the questionnaire before completing it, and to the anonymous data being collected and used for the evaluation of group and to be shared through publications, social media and with other clinical centres.

#### Questionnaire administration

The questionnaire was made available to members of the PPIE group using Microsoft Forms and was open for completion for two weeks in May 2023.

#### Questionnaire analysis

Once the questionnaire was closed to new responses, a researcher (KH) completed an initial review and analysis of the data before sharing it with the group. Two thirds (2/3) of the members of the steering group responded to the description of the raw data, which included an interpretation of the free-text responses. Analysis of the rating scale questions using simple descriptive statistics, counts and percentages, and reflexive thematic analysis [27–29] of free-text answers was completed by a researcher (KH). KH is a researcher and co-lead of the PPIE group and has been involved in the development and running of the group from the start. Building interpersonal relationships with group members over time and feeling some responsibility for the success of the group likely influenced how the data were interpreted during the thematic analysis. The data were shared with group members to provide feedback on/to discuss findings before writing up.

**Fig. 1:**
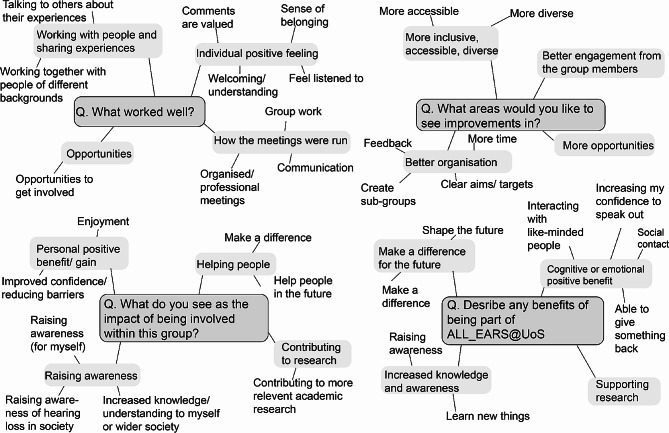
Reflexive thematic analysis process (phases one to three). The free-text questions (dark grey box) and the initial themes developed for each question (light grey boxes) and initial codes are included around each theme

The reflexive thematic analysis followed Braun and Clarke’s six phase process [27–29], (1) Familiarising yourself with the dataset (2) Coding (3) Generating initial themes (4) Developing and reviewing themes (5) Refining, defining, and naming themes (6) Writing up [30]. This approach is flexible but robust and an established analytical tool. The aim was to determine what areas are important for the group. Inductive coding was used whereby there were no pre-determined codes. A set of codes were developed based on the review (reading and rereading) of the data. The initial review of the data was completed by reading and extracting the quotes or phrases (phase one). These were pasted into a spreadsheet. Initial codes were developed from the individual responses and each response in a row of the spreadsheet was assigned to each of the codes (phase two). From these initial codes, initial themes (light grey boxes) were developed (phase three). Figure 1 is a diagram highlighting phases one to three.

**Fig. 2:**
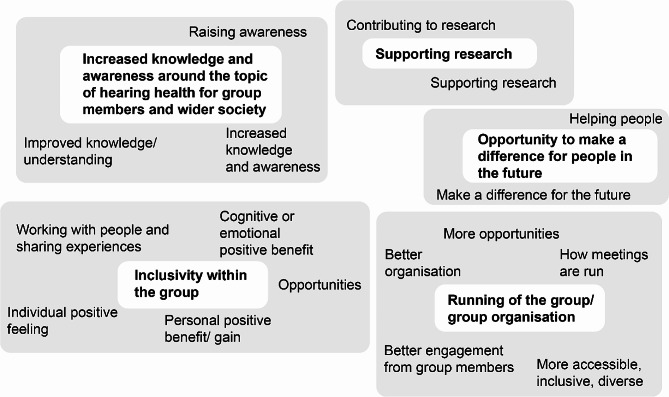
Phase four and five of reflexive thematic analysis. Phases four and five include developing and reviewing themes (four) and then refining, defining, and naming themes (five). The initial codes from each open-text question was assigned to initial themes which are included in the grey box above. These were developed and reviewed during phase four and then refined and assigned into five refined themes (highlighted in the white boxes with bold text) during phase five

The initial themes (Fig. 1) were developed, reviewed and refined to give five themes (indicated in bold in Fig. 2). An audit of the process, a record of all raw data and detailed notes of the analysis process including personal reflections were completed to ensure data trustworthiness [31–33]. During phases three and five, diagramming was used to make sense of how initial codes would develop into initial themes that could be developed further (Figs. 1 and 2). During phase four, themes and subthemes were discussed with team members and refined. The final phase (phase six) to write up the data was done by KH and shared with the rest of the team for feedback.

The findings of the work, and the structure and content of this paper, have been formatted to align with the GRIPP2 Long Form [34] [see additional file 1].

## Results

The results are split into two main sections. The first section reports the data collected through monitoring the engagement and activities of ALL_EARS@UoS between May 2022 and September 2023. The second reports the findings from the evaluation questionnaire that was co-designed and sent to members of ALL_EARS@UoS, after one year of running the group (May 2023).

### The engagement and activity of ALL_EARS@UoS

#### Group members/attendance

Eight in-person meetings were held between May 2022 and September 2023, with attendances of between five and 14 people. Table 3 summarises attendance at each meeting alongside how people came to join the group. Name and number of attendees for each meeting is recorded, no identifiers are used in any of the reports.

**Table 3:** Breakdown of number of attendees for each meeting held

Number of meetings	Date of meeting	Total attendance	Breakdown of attendees			
			New members	Patients at USAIS	Recruited through adverts (not patients)	Recruited from local community
1	03/05/2022	9	9	8	1	0
2	22/06/2022	5	1	5	0	0
3	23/09/2022	6	2	5	1	0
4	23/11/2022	7	0	6	1	0
5	06/01/2023	12	6	9	3	0
6	10/05/2023	14	10	4	0	10
7	11/07/2023	14	3	5	0	9
8	18/09/2023	12	1	3	0	9

Data summarising the number of meetings held, attendees and route by which people joined the group (e.g. patients at USAIS or through community engagement)

#### Supporting research

Eight distinct research projects have benefitted from contributions of ALL_EARS@UoS members. This has included input to the design and implementation stages of projects. Member input was through the discussion of project ideas in group meetings and critique and additions to documents. This included project outline summaries, governance and patient and participant facing documents including participant information sheets that were shared with group members to review and comment on. One group member contributed to the panel meeting for external ethical approval by the UK’s Health Research Authority. Group members have contributed to three research grant applications by providing feedback on the rationale and their perceived need or value of the work, review of applications and by providing letters of support. Three group members contributed to a video submission explaining the methodology for an application to a national funding body.

Through learning about group member’s experiences of getting their cochlear implant and the challenges some faced, a new project to evaluate the barriers and facilitators to cochlear implantation in a population of patients from USAIS who are over 60 emerged. The findings from this study have been presented at a national hearing meeting [35] and are now being written up for publication in a peer-reviewed journal.

#### Public engagement

Members of ALL_EARS@UoS have been involved with a diverse range of public engagement activities and events. In July 2022, a member of the group and a researcher gave a joint presentation reflecting on the process of the developing the group at an event called Working together: A training workshop on participatory and co-produced research. We have jointly staffed an exhibit about hearing and cochlear implants at University of Southampton Science and Engineering Festival in March 2023 and seven members of the group volunteered their time to run the exhibit alongside researchers and raise awareness of hearing loss to members of the public of all ages. We took the same exhibit to the New Forest and Hampshire County show in July 2023, where three members of the group ran the exhibit alongside the researchers.

### Evaluation questionnaire findings

#### Demographic information

The response rate for the survey was 61% (11/18) of the PPIE group members. All respondents were over the age of 30 years old. Table 4 summarises the age, sex, and ethnicity of the ALL_EARS questionnaire respondents alongside age, sex, and ethnicity of that of USAIS patient population and of South of England. The majority of questionnaire respondents were aged 50–79 years old (73%, (8/11)) and white (91%, (10/11)). 45% (5/11) of respondents described themselves as ‘Retired with several community commitments e.g. ALL_EARS, member of school or other community board’. 36% (4/11) of respondents described themselves as employed, two of whom work full-time and two part-time. 18% (2/11) respondents described themselves as ‘Retired with one community commitment e.g. ALL_EARs’. All respondents selected their primary/native language as English.

**Table 4:** Age, sex, and ethnicity data for respondents, USAIS patient population and the South of England

Group		ALL_EARS questionnaire respondents	USAIS Patient Population ^a^	South of England ^b^
Age	Under 30	0%	26%	34.5%
	30–50	9%	23%	26.2%
	51–80	73%	45%	34%
	Over 80	18%	5%	5.3%
Sex	Male	36%	40%	
	Female	64%	60%	
Ethnicity	White	90%	90%	89%
	Black	10%	5% (5% no data for)	1.9%
	Asian	-		5.4%
	Mixed	-		2.5%
	Other	-	-	1.2%

^a^ Data were extracted from a service evaluation of data from USAIS electronic patient records of all patients at USAIS who received a cochlear implant until 2020. (ERGO II: 76,664)

^b^ Data were extracted from 2020 ONS Clinical Commissioning Group (CCG) population estimates [36] and the UK census 2021 databases [37]

The demographic of the group at the time of the survey was similar to that of the patient population under the care of USAIS; the majority of USAIS patients describe themselves as White (British) or White (any other white background) (90%). Of the USAIS patient population, the largest proportion of people are aged between 50 and 80 years old which reflects the age distribution ALL_EARS@UoS. Current data for our primary clinical catchment area identifies that around 89% of people are White, 5.4% Asian, 1.9% Black, 2.5% Mixed and 1.2% Other. A similar assessment of the population by age shows 34.5% of people are under 30, 26.2% are aged 30–50 years old, 34% of people are aged between 51 and 80 years and 5.3% of people are over the age of 80 years. We aspire to develop the demographic of our group to more closely reflect the age and ethnicity of the South of England, the catchment area of the clinical centre.

A question was included to determine whether respondents had prior knowledge of deafness or the deaf community before developing hearing loss (or a family member/partner having hearing loss). 78% of respondents (8/11) reported no prior knowledge.

#### Rating scale questions

**Fig. 3:**
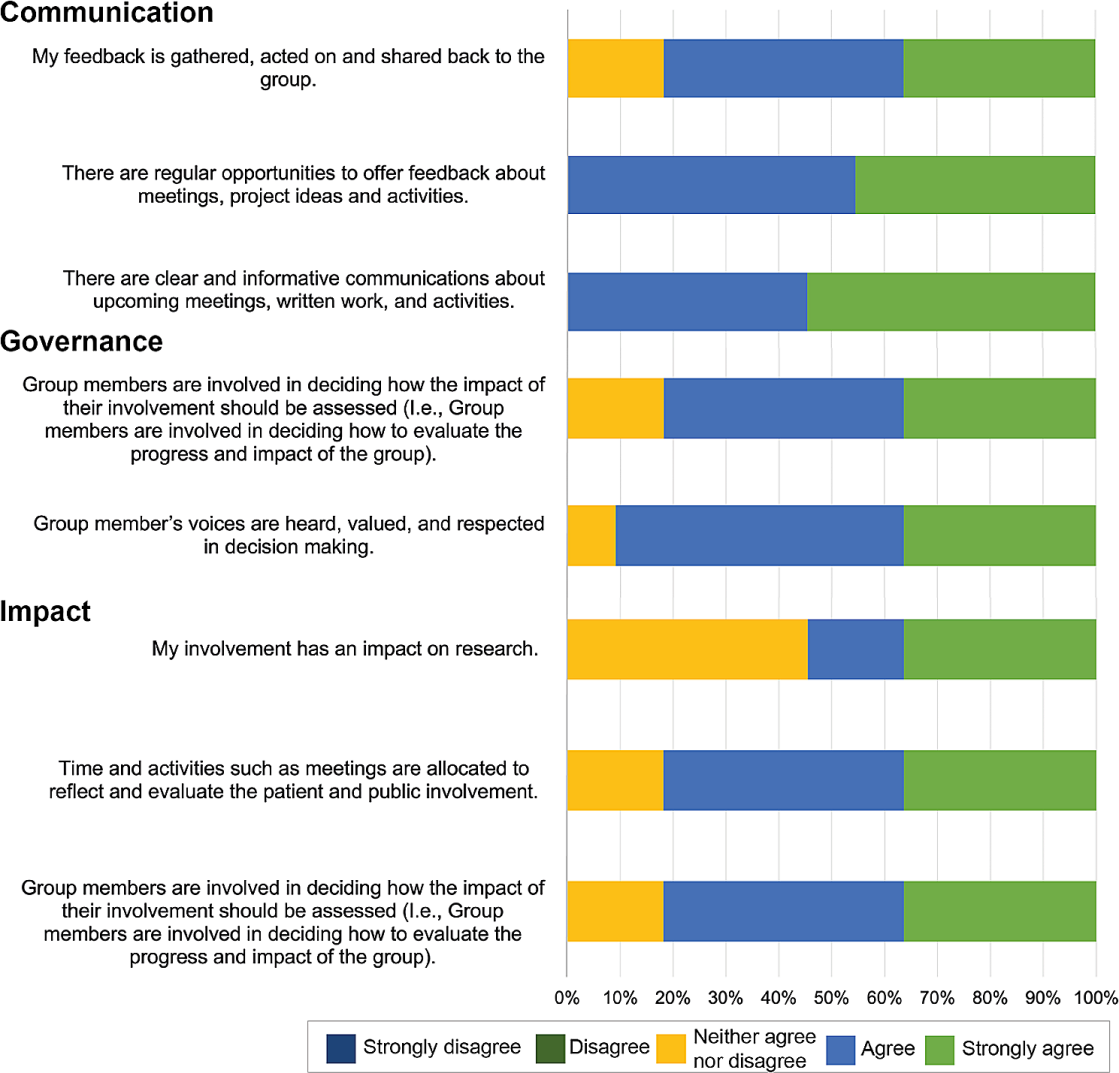
Responses from rating scale questions that address the UK Standards for Public Involvement. The standards include communication, governance, and impact

**Fig. 4:**
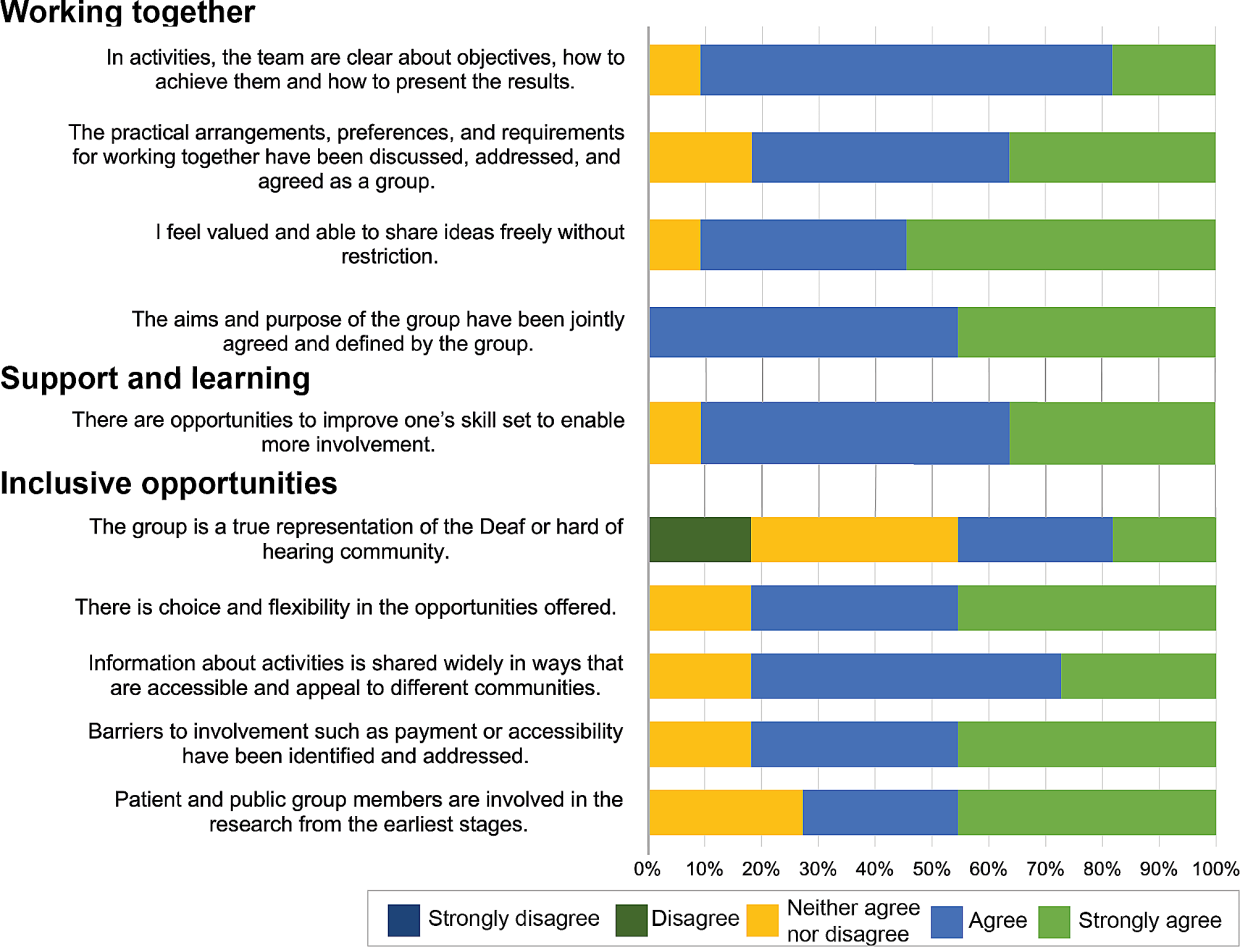
Responses from rating scale questions that address the UK Standards for Public Involvement. The standards include working together, support and learning and inclusive opportunities

There was a positive response, *strongly agreed* or *agreed*, to most rating questions, summarised in Figs. 3 and 4. Figure 3 displays the statements included for Communication, Governance and Impact and the percentage of respondents that selected each response. Figure 4 displays the statements included for Working together, Support and learning and Inclusive opportunities and the percentage of respondents that selected each response. In Fig. 3, the statement about ‘clear and informative communications’ achieved the highest rates with all responses being strongly agree or agree. Whereas the statement ‘my involvement has impact on the research’ had the lowest rates (only 50% were strongly agree or agree). In Fig. 4, the statement about ‘the aims and purpose of the group have been jointly agreed and defined by the group’ achieved the highest rates. The statement ‘the group is a true representation of the Deaf or hard of hearing community’ scored the lowest rates with under 50% of responses being agree or strongly agree. A few responses were neither agree nor disagree, suggesting ambivalence and that more work needs to be done to enable the group to agree that these standards are being met.

#### Reflexive thematic analysis of open-text questions

Five themes were developed from the dataset (Table 5) which include: (1) Increased knowledge and awareness around the topic of hearing health for group members and wider society, (2) supporting research, (3) inclusivity within the group, (4) opportunity to make a difference for people in the future and (5) running of the group/group organisation. Two themes are comprised of subthemes.

**Table 5:** Themes and subthemes

Theme	Subtheme
1. Increased knowledge and awareness around the topic of hearing health for group members and wider society	1.1 (Increased) knowledge to individual and society 1.2 Raising awareness (of hearing loss and cochlear implants) for the individual and society
2. Supporting research	
3. Inclusivity within the group	
4. Opportunity to make a difference for people in the future	
5. Running of the group/ group organisation	5.1 Effective communication and organisation within the group 5.2 Suggestions for organising the group

### Theme 1: increased knowledge and awareness of hearing health for group members and wider society

This theme captures how through being involved with the group, members felt they had gained greater knowledge and awareness, or had contributed to wider society growing in knowledge and awareness, of hearing health.

#### Subtheme 1.1 (increased) knowledge to individual and society

Group members commented that the opportunity to learn new things was a positive outcome of the group ‘increased (my) understanding of research and how it all works [P3]’ as well as the opportunity learn from the group to then pass on the ‘important information for my community [P10]’, particularly around ‘what help is offered [P5]’.

“I learn new things about cochlear implants and hearing loss whilst helping to raise awareness [P2]”.

“It has been helpful to find out more for my chats to others with hearing loss as part of the bigger picture [P9]”.

Some group members discussed the need for further learning/education in ‘understanding the practical difficulties of being hard of hearing and elderly [5]’ and in ‘explaining signal processing aspects of deafness [P7]’.

#### Subtheme 1.2 - raising awareness (of hearing loss and cochlear implants) for the individual and society

This subtheme has two main elements; the need for increased awareness (which the group aims to address) and the positive impact of the group in increasing awareness of hearing loss and cochlear implants.

Five responses emphasised the need for increased awareness around hearing loss and the impact of hearing loss on the individual, as well as a need for much better awareness of cochlear implants. This appears to be a driving force for the involvement of many members of the group. A key reason why we need to raise awareness was to give people a better understanding of the challenges people with hearing problems experience ‘to help people understand the problems that are faced by the deaf community [P5]’.

“Raising awareness of hearing loss so people have a better understanding of it and take into consideration how it can affect people [P2]”.

Better awareness and knowledge of cochlear implants will be key in increasing access to cochlear implants, which is a shared driver of our group. One respondent discussed the importance of ‘reaching out to deaf people and parents of deaf children who know little or nothing of cochlear implants and their availability [P6]’.

Three responses highlighted the positive impact of the group in raising awareness of hearing and cochlear implants for themselves and in wider society.

“Raised my awareness of the barriers some people face in terms of getting a cochlear implant and also our work has raised awareness of hearing issues within wider society [P3]”.

“spreading the word about implants particularly to older people through going out to groups [P9]”.

Two respondents looked forward to further impact of the group in continuing to raise awareness as the group develops and builds in the future.

“There is certainly scope for the group to have more impact on wider society as it develops in numbers and confidence [P9]”.

“I hope our impact will develop as we provide opportunities to further education on hearing loss (UoS Science Open Day and Winchester Science Centre) [P7]”.

### Theme 2 - supporting research

Three responses reflected the perceived benefit or positive impact of the group in ‘supporting research and development opportunities [P8]’ which would then go on to benefit those with hearing loss or wider society.

“Difficult to know but I’d like to think that the work we’ve done collectively has helped in terms of creating research projects which in turn will have an impact on those with hearing loss [P3]”.

“The opportunity to contribute to research topics which will, in turn, benefit wider society [P4]”.

A group member discussed the opportunities to contribute to research by ‘suggest(ing) changes to formal documents to better reflect the lived experience’ as well as offering ‘the academic members suggestions for the thrust of their work [P11]’.

One group member described it to be ‘stimulating to be visited by members of the research community e.g. Tracey and Helen and invited to comment on and add to their plans [P9]’.

### Theme 3 – inclusivity within the group

Two group members highlighted the feeling of being valued and listened to, after years of potentially not feeling listened to (due to their deafness).

“I’ve been losing my hearing for 20 + years and this is the first time anyone has asked me for my opinion. It genuinely feels like we’re listened to, and our comments are valued and appreciated. It feels like the group is building momentum, and going forward has the capacity to have an impact and make a difference [P3]”.

“Encouragement to speak out and listen, using my implant to its full ability. The confidence to participate in discussions after many years of being mostly excluded from conversation [P6]”.

Two group member’s responses indicated a sense of good, a welcoming feeling towards all members of the group and discussed a feeling of belonging. This aligns with UK Standard ‘Working together’ which emphasises working together in a way that values all contributors and builds and sustains mutually respectful and productive relationships.

“To be in a group of your peers with researchers who are very understanding and encouraging is very fulfilling [P9]”.

“All the preparation work, and keeping patient involvement in that, has given us all a sense of belonging and an aim of making the group a success [P4]”.

There is a positive feeling within the group due to the ‘common goals’ and collective good wishes.

“The company and interactions of people with similar or complementary wishes for the deaf community [P7]”.

In addition, four responses highlighted the benefits of sharing experiences with a group of people, and ‘talking to other people about their journey of hearing loss and a cochlear implant [P2]’ and the benefits of ‘getting a wider diversity of people involved [P9] and ‘getting people from different backgrounds to work together [P7]’’. This links to the inclusivity aspect of theme 5. One member commented on their ‘improved my confidence in terms of getting involved with things [P3]’.

### Theme 4 – opportunity to make a difference for people in the future

Four responses highlighted the intent of the group in making a positive difference for people who may experience deafness in the future. One member felt they wanted to ‘be part of changing things for the future, and (feel) that the group provides that opportunity [P3]’.

“Make a difference in my community and encourage people to get involved [P10]”.

“I have had a lifetime of involvement with severely deaf people, throughout which I have seen well-intentioned but sub-optimal efforts to educate them and care for their welfare. I hope that and my technical skills can be used to improve their lot and help those who will become deaf in the future [P7]”.

One member felt the group provided an ‘opportunity to shape the future of hearing services, medical studies and outcomes [P4]’.

### Theme 5 - running of the group/group organisation

Fifteen responses related to the organisation and structure of the group, where members of the group commented on either positive aspects of the organisation of the group or suggestions of how the organisation and structure of the group could be improved. This theme splits into two subthemes which include:

#### Subtheme 5.1 effective communication and organisation within the group.

Feedback suggests members of the group are satisfied with the ‘communication [P5]’ and the structure and organisation of the group. One response said, ‘The arranging of meetings and communications about what the targets for the meeting are done professionally [P11]’.

“The meeting I attended was very well structured [P10]”.

“The discussion groups have worked well where we go into small groups and then bring out ideas together at the end [P2]”.

#### Subtheme 5.2 suggestions for organising the group

There were eleven suggestions for how we could improve the organisation or running of the group. These related to a variety of areas including structure, inclusivity, and level of involvement. Suggestions for improving the structure referred to practicalities for how the meetings are scheduled and organised and how long the meetings are run.

“From my perspective, I’d like to have some meetings scheduled in the calendar for the coming year [P3]”.

“An extra half hour added to the meetings, so the agenda isn’t so rushed at the end [P4]”.

“Possibility to create smaller subgroups to focus on specific things [P8]”.

As we are a group with people with lived experience of hearing loss, ensuring all members of the group have equal access to the information and discussion during, and after, the meetings is always carefully considered. We only hold our meetings in a room with suitable lighting and space, with microphones connected to a loop system. We provide live captioning in each meeting. Several comments around improving accessibility were related to the times and format of the meetings i.e., whether they are online or in person. To date, we have only held in-person meetings. However, one response highlighted the potential benefit of holding some online meetings which will be included in our action plan. ‘We talked at the beginning about meeting times and whether in person or video. I may be the only person who has missed a couple of meetings but would like to ensure I can be present, so I contribute effectively [P8]’. In addition, up until now, we have held meetings from 10am – 12 noon, following a survey given to members during the early stages of the group which indicated a preference of mid-morning meetings. However, mid-morning meetings limit some people from attending including those who work full-time. A suggestion from a group member was that ‘The meetings could (to) be more accessible for people who work. This could also increase the number involved in the group [P2]’. These responses align with the UK Standard ‘Inclusive Opportunities’ which states that public involvement opportunities should be accessible and should reach people and groups according to research needs [22].

A further suggestion for ways we can improve the organisation of the group included better and more complete feedback during and following involvement. ‘Where members have been or are being involved, it would be good for others to get feedback on how things are going and where things have changed as a result [P11]’. It is key in PPI to complete the feedback cycle, so group members/public contributors know the impact of their involvement and to know that their thoughts/opinions have been acknowledged and listened to. One response highlighted the importance for **‘**more opportunities to collaborate and share outputs, data and insight from the work to educate and drive continuous improvement in this field [P8]’.

### Level of involvement

One response suggested there may be an imbalance regarding expectations for what a group member’s level of involvement should be and that group members should be prepared to get involved further with various tasks and meetings.

“Those who attend the group, in whatever way, being prepared to respond to requests to be involved in research e.g. through questionnaires, structured interviews etc. as well as discussion. I can quite understand that being involved in being a lay responder to research funding requests might well be too daunting for many. It would be helpful if more people were prepared to come to more meetings so people can develop confidence in working as a group. I quite understand it is early days for this at present [P9]”.

#### Feedback regarding governance

Three responses agreed the questions posed about Governance were ‘good questions [P4]’. One response highlighted that ‘If we choose to comply with a set of guidelines or standards, we should say so and should note that we do this because it makes the research more effective (this may be as simple as making the findings more acceptable to the intended audience) [P1]’. Two responses acknowledged the effort and resources we have put into this work ‘Looking at the development of the membership of All Ears it is obvious that great efforts have been made to involve a broader range of people [P9]’ and ‘At the All_Ears level, there is a great feeling that finance and resources are being put into genuine PPI [P11]’. A response suggested there was some uncertainty around ‘where All_Ears sits in relation to PPI in other branches of The University and the Southampton Hospital/Medical School. Thus I am uncertain where the thrust to promote the approach is coming from [P11]’. This suggests further discussion and transparency is required with our members about the structures in place to support the PPI.

## Discussion

This paper evaluates the impact, effectiveness, and engagement of the PPIE group ALL_EARS@UoS using the UK Standards for Public Involvement as a framework. Overall, the strongest satisfaction was seen in responses to the statements related to communication and working together. Five themes were developed, these centred on increasing knowledge and awareness around hearing loss and cochlear implants, supporting research, the positive impact of the group on the individual as well as for wider society and how we organise the group. The responses suggest members value being part of the group and feel valued by researchers and other members of the group. Two areas requiring improvement and attention are increasing and improving diversity, and ensuring inclusivity within the group. Our evaluation findings overlap with the seven themes identified in a systematic review and thematic synthesis of the experience of patient partners in public involvement [38]. These included “motivations to engage in research”, “activities in patient engagement”, “structure”, “competence”, “team dynamics”, “impacts on broader life”, and “illness”. ‘Structure’ and how the PPI is organised was another key area of importance which reflected both positive and negative patient experiences. This was also found in our evaluation. The importance of effective communication is a commonly discussed area in PPI evaluation [39–41].

The rationale for using the UK Standards for Public Involvement to structure this evaluation [24–26] was that we could then use the same structure to form the basis of the action plan that would emerge from the evaluation. We have used the evaluation as an opportunity to inform group members of the UK Standards for Public Involvement to ensure we are being transparent about the expectations of effective PPIE. We have always aimed to be upfront and honest with our group members about the aims and intention of our PPIE work including the standards we aim to align to. Further adoption of the UK Standards for Public Involvement would facilitate the comparison of successes and challenges across regions, nationally and internationally. A limitation of our evaluation was that we did not have a version of the questionnaire for researchers/PPIE team to complete to evaluate the impact, effectiveness, and engagement of the group from researchers’ perspective. Going forward, this will be essential to implement. Another evaluation tool available is the Public and Patient Engagement Evaluation tool (PPEET). This is a selection of anonymous questionnaires developed collaboratively by researchers, patients, and members of the public [42, 43]. There are three types: a participant questionnaire, a project questionnaire, and an organisation questionnaire. (Note. in Canada, patient and public involvement is referred to as patient and public engagement). PPEET was developed as an evaluation tool that is user-friendly, generic and aspires to standardise evaluation. The international standardisation of this approach and the availability of both a pre-designed participant questionnaire and a project questionnaire for researchers and PPI professionals to complete are both strengths of this tool. We will consider this for future evaluation.

### How valuable?

The value of embedding PPIE into the work we do has been significant and wide reaching. We have had beneficial contributions to eight research projects (ranging from student-led projects to a larger-scale bid) where members have been involved in the design stages of the projects including reviewing project documents. This has improved the clarity, readability, relevance, and content of the documents. Further benefits of PPI in the design stages of hearing research have been described which includes increased participant recruitment [17] and creating new research ideas [16]. A limitation of our PPIE is that we were unable to involve group members in the ideas/planning stage of some projects due to time constraints and short windows for grant funding calls.

For members of the group, there has been great personal value and impact as highlighted in the qualitative data collected in the questionnaire. We have created a group where there is mutual trust, and members feel valued and empowered to share their experiences as evidenced through group member’s involvement in public engagement events like at the University of Southampton Science and Engineering festival. Truly valuing group members unique knowledge and lived experience, and how this will contribute to the research, is essential [44]. A variety of positive outcomes of being involved in research on the public have been described in the literature including new skills and knowledge, personal development, and new support/friendships [2, 4].

PPIE is valuable for researchers and research students in developing their effectiveness and expertise in research and science communication [45]. It provides an opportunity to develop interpersonal skills and the skills required to organise and facilitate meetings and events. We have seen this first hand in our research group. Importantly, PPIE allows researchers to learn about their research area from the perspective of people with lived experience [4] which enables the development of a deeper understanding, often otherwise largely theoretical, of a health condition. To gather data on the perspectives of researchers in our group, we will evaluate the value and the challenges using a similar questionnaire as described in this article.

Through establishing ALL_EARS@UoS PPIE group, we have been able to bridge the gap between the university and community by working with members of our local community and learning from their knowledge and experience. This is a priority at University of Southampton as we are part of a Civic University Agreement [46] meaning we are committed to improving the lives of those living in our local area by engaging with them through different educational, enterprise, and cultural activities. Through going out into our local community, we have successfully recruited several people to join our group, increasing their opportunity to be heard and the cultural and ethnic diversity of the group. Co-designing and co-delivering exhibits at public engagement events with members of the group, has developed new ways for us to share knowledge about our research with members of our local community.

### How hard?

Involving a diverse group of people who will represent different communities is essential for PPIE to be truly effective. Many groups in our society are considered underserved or ‘seldom heard’ [47, 48]. The demographic of our group at the time the evaluation was not very diverse (majority white and over the age of 50) and most group members were recruited through being a patient at USAIS. We were aware this was not representative of our local area as well as not a diverse group of people in terms of their experience of hearing loss. We need to increase our efforts to make the group relevant to people under the age of 30 as people in this group are also affected by hearing loss. We have used community engagement as a method to break down physical barriers by going to people where they are in the community and by investing time to build trust. Through visiting local community groups and getting to know community members by getting involved with activities; we have been building trust and creating a more equal partnership which is key for effective involvement and engagement. A similar approach [49] that has a clear focus on how to build trust and relationships, conduct acceptable and inclusive activities and maintain reciprocal relationships has been discussed.

At the time of writing, 40% (16/40) of our group members have been recruited through community engagement, helping to make the group more representative of our local community. We now have members who have different types of hearing loss, who wear hearing aids or cochlear implants (or both or neither), as well as people who are interested and want to support their families and communities by being involved with hearing loss research. Further improvements are needed with regards to how diverse the group is within deafness. This was highlighted in the questionnaire rating scale question ‘The group is a true representation of the Deaf or hard of hearing community’ (Fig. 3). We currently do not have any members from the Deaf community who communicate solely by sign language, who regularly attend our meetings. Over time, when we re-evaluate the group, we anticipate a more diverse demographic and a higher number of responses, due to the gradual increase in group attendance.

Diversity and inclusivity should come hand in hand. Having diversity within PPIE without inclusivity is poor. Inclusivity means a combination of acknowledging and valuing differences whilst also promoting equity. Inclusion is as an active verb; something we must *do* to ensure people have equal access to the information, meetings, and group. We have invested considerable effort to create an inclusive group and ensure we are meeting each member’s accessibility needs, which has been a challenge. Our group members have different levels of hearing loss and use a variety of hearing interventions and communication approaches. For communication between meetings, all members were asked to share their communication preferences when they joined the group such as via email, phone calls or text messaging or letters. In each meeting, we have a note taker present and live captions are projected onto a screen, which allow members to glance at the screen and follow what is being said. The rooms we use have multiple screens to allow for projection of live captions as well as any slides for the meeting. The rooms have a hearing loop system which is connected to the microphones we use. If we have any members who attend our meetings who communicate using British Sign Language (BSL), we will have a BSL interpreter present. A significant challenge is the cost to support these accessibility needs which are on top of the cost of involvement fees [50] and travel expense that are offered to all participants at each meeting.

Our monitoring data, shows an increase in the number of members attending each meeting as well as an increase in the number of members in the group. A regular funding source is necessary to support and sustain the PPIE activities. As well as accessibility costs, funding is required to pay for involvement fees and travel expenses [50], staff time [51], materials and refreshments. PPIE needs to be accurately and appropriately costed into every research grant. A systematic review exploring the theories, barriers, and enablers of PPI across health, social care and patient safety identified financial compensation and resources as a key area to consider [52]. There is an underreporting of the costs related to PPI and evidence of any economic analysis of the costs in the literature [3, 4, 53, 54]. The importance and challenges of carrying out cost evaluations of PPI have been discussed [55]. Using established economic evaluation methods for PPIE has limitations as outcomes for PPI cannot be expressed as quantifiable entities [55]. In addition, we cannot solely focus on outcomes of PPIE, as the process including the trust and relationships formed are equally as important. This is likely contributing to the apparent disconnect or lack of understanding of the true costs or budgets that are required for inclusive and enduring PPIE by both funding bodies and researchers.

Maintaining effective communication with our group members was identified as a strong area in both the rating scale questions and free-text answers. Communication is one of the six UK Standards for Public Involvement which states the importance of plain language for well-timed and relevant communications, as part of involvement plans and activities. Building trust and relationships with group members and maintaining effective communication all take time which can be a challenge for researchers. Having one main PPIE contact or lead for consistency, and to build the relationships is documented as being important [51, 56, 57]. However, this can also be challenging in academic settings, where many researchers are on short-term contracts. The time required to build and maintain enduring PPIE groups should be recognised in academic settings to ensure the appropriate funding and support is in place.

### Is PPIE being taken seriously?

Responses from the questionnaire highlight that ALL_EARS@UoS members recognise the value of the PPIE in raising awareness for hearing loss and supporting research. This is echoed in the literature [13, 58, 59]. Despite this, and the growing need expectation of PPIE, it can be seen or perceived as something less important than doing the ‘real’ science research [60, 61]. Therefore, it has been challenging as a group of pre-clinical science researchers who are trying to do meaningful, sustainable PPIE. Considerable staff time, planning and resources have been invested in establishing our PPIE strategy and building the group, as we recognise the need and true value of PPIE. A continued challenge in pre-clinical research will be to increase awareness of the importance of PPIE. Could this happen at an institutional level and/or are national funding panel members adequately trained to assess the robustness of PPIE within funding bids?

### Looking forward

Using the findings from our evaluation, we have produced a PPIE action plan for the next 18 months and beyond [see additional file 3]. Actions, which align with each of the six UK Standards for Public Involvement, have been developed using the data from the evaluation, alongside data we collected through ongoing monitoring. These will be reviewed and evaluated 18 months from their start date.

We have seen the benefits of going out into our local community, engaging with a broad range of people, and building trust. We will continue to build and develop our community links ensuring we reach communities/groups that are less well served. This will address the local health inequalities related to hearing health by increasing access to information and services. Through building trust and relationships with community members, we aim to increase participation and involvement with research. An idea that stemmed from our group is to have ‘community ambassadors’ from ALL_EARS@UoS who are equipped to go into the community to raise awareness. Community ambassadors will use their existing links to widen their reach in the community. Working together we will build upon the work we have started in the community and widen participation and involvement in our research and PPIE to ensure that more voices are heard.

## Conclusions

We have presented findings from the ongoing monitoring of ALL_EARS@UoS activity, alongside an evaluation questionnaire and discussed how valuable and how hard PPIE can be for both group members/participants and researchers. The value of PPIE for our group members includes increased knowledge, feeling valued and listened to, sharing experiences with others, and supporting research. A challenge for group members is being involved alongside other commitments such as caring responsibilities and the demands of employment. We intend to address this through a focused effort to run online meetings at various times during the day/out of core work hours. The value of PPIE for researchers in the group has included learning from people who have lived experience of hearing loss and what matters to them, developing new ideas for projects with group members and developing their professional and interpersonal skills. A challenge for researchers is maintaining a regular funding stream to sustain the group, as well as the time and expertise required to run an effective group. Increasing the visibility of our group and highlighting the need for support from local, and possibly national, researchers through funding bids are ways we are tackling this. Finally, ensuring PPIE is both diverse and inclusive remains a challenge to all involved. These aspects require consistent commitment to improvement from researchers and PPIE practitioners. We are working to achieve and maintain this by going to people in the places where they are and engaging with different communities, particularly those seldom heard voices to ensure they too can share their lived experience.

### Electronic supplementary material

Below is the link to the electronic supplementary material.

**Supplementary Material 1:** GRIPP2 Long Form checklist

**Supplementary Material 2:** ALL_EARS@UoS Evaluation Questionnaire

**Supplementary Material 3:** ALL_EARS@UoS PPIE Action Plan

**Supplementary Material 4:** Framework for reviewing documents

## Data Availability

The dataset supporting the conclusions of this article is available in the University of Southampton PURE repository, 10.5258/SOTON/D2919.

## Declarations

### Authors’ information

**KH** is a post-doctoral research fellow in Faculty of Medicine and PPIE group co-lead for ALL_EARS.

**MG** is a senior clinical scientist at University of Southampton Auditory Implant Service.

**HP** is a PPIE (Patient and Public Involvement and Engagement) lead and advocate.

**WBJ** is a patient and public involvement practitioner.

**SS** is a member of ALL_EARS and has a cochlear implant.

**CS** is a member of ALL_EARS and has a cochlear implant.

**IH^** was a member of ALL_EARS who was involved in the design stages of the evaluation.

**ST** was a member of ALL_EARS who was involved in the design stages of the evaluation.

**TN** is a Professor in Neuroimmunology and Director of the Graduate School for Faculty of Medicine.

## Abbreviations

BSL: British Sign Language
IRAS: Integrated Research Application System
NIHR: National Institute of Health and Care Research
PPEET: Public and Patient Engagement Evaluation tool
PPI: Patient and public involvement
PPIE: Patient and public involvement and engagement
UKRI: UK Research and Innovation
USAIS: University of Southampton Auditory Implant Service
