# Supplementary material for: Patient and public involvement and engagement (PPIE): how valuable and how hard? An evaluation of ALL_EARS@UoS PPIE group, 18 months on
Source: Res Involv Engagem. 2024 Apr 11;10:38. doi: 10.1186/s40900-024-00567-1 (PMC11010367; doi:10.1186/s40900-024-00567-1)
Supplement: Supplementary file 2 — Supplementary Material 2: ALL_EARS@UoS Evaluation Questionnaire [file 40900_2024_567_MOESM2_ESM.docx]

**Patient and Public Involvement and Engagement Evaluation – ALL_EARS@UoS**

**Introduction:**

It is a year since we first advertised for people with lived experience of hearing loss and cochlear implants to come and meet some researchers. This grew to become ALL_Ears@UoS. We have held 5 meetings since then, had various engagements with the wider community and now have 21 members in the group.

As the group reaches its 1^st^ birthday, we thought we should reflect on and evaluate what has worked well and what we need to improve. The evaluation questions are based on the Guidance for reporting involvement of patients and the public (GRIPP2) framework, a tool used to evaluate and report PPI. The combination of short and longer answer questions should enable us to collect the breadth of information needed.

The data will be used to evaluate what has happened so far and to produce an action plan for the coming 12 months and beyond. The questionnaire is anonymous, this is so that we can share our findings with other support groups, clinical centres, and researchers. This will include a peer reviewed research paper which will describe the development of All_Ears@UoS, our progress and our future aims and goals.

This work will ensure that All_Ears@UoS is relevant for its members and will be used to support continued funding for this activity.

**Please tick this box below to confirm the following:**

You are happy to participate in this research.

You are happy for the data collected to be used for evaluation of ALL_EARS@UoS PPIE group.

You are happy for the fully anonymous data collected to be shared through publications, social media and with other clinical centres.

As this is an anonymous questionnaire, it is not possible for participants to withdraw their data retrospectively.

**Demographic data**

This data is so we can evaluate the reach of All_Ears@UoS relative to the population of people at USAIS and in the geographical region covered by the clinic.

**Which category below includes your age?**

17 or younger

18-29

30-49

50-69

70-79

80 - or older

**What is your gender?**

Female

Male

Non-binary

Other (please specify)

**Ethnicity**

**What is your ethnic group? Choose one option that best describes your ethnic group or background.**

**Asian or Asian British**

- Indian
- Pakistani
- Bangladeshi
- Chinese
- Any other Asian background

**Black, Black British, Caribbean or African**

- Caribbean
- African
- Any other Black, Black British, or Caribbean background

**Mixed or multiple ethnic groups**

- White and Black Caribbean
- White and Black African
- White and Asian
- Any other Mixed or multiple ethnic background

**White**

- English, Welsh, Scottish, Northern Irish or British
- Irish
- Gypsy or Irish Traveller
- Roma
- Any other White background

**Other ethnic group**

- Arab
- Any other ethnic group

**Employment**

**Which of the following categories best describes your employment status?**

Employed, full time

Employed, part-time

Full time carer

Not employed, looking for work

Not employed, NOT looking for work

Retired – with several community commitments e.g. All_Ears, member of school or other community board

Retired – community commitment All_Ears

Disabled, not able to work

*If you are a combination of the above categories, please list the categories below.*

………………………..

**What is your primary/native language?**

**Did you have any prior knowledge of deafness or the deaf community?**

The National Institute for Health and Care Research (NIHR) is the British government’s major funder of clinical, public health, social care, and translational research. It is the nation’s largest funder of health and care research, providing the people, facilities, and technology for research to thrive.

**The NIHR have an established set of National Standards for Patient and Public Involvement which cover six areas. The standards are below.**

**Communications:** use plain language for well-timed and relevant communications, as part of involvement plans and activities.

**Governance:** involve the public in research management, regulation, leadership, and decision making

**Impact:** seek improvement by identifying and sharing the difference that public involvement makes to research

**Working together:** work together in a way that values all contributions and builds and sustains mutually respectful and productive relationships.

**Support and learning:** offer and promote support and learning opportunities that build confidence and skills for public involvement in research.

**Inclusive opportunities:** offer public involvement opportunities that are accessible and that reach people and groups according to research needs.

Using these standards, we have put together some questions to get an idea of how we are doing in each area of the standards.

***Please indicate how strongly you agree or disagree with the following statements.***

**Communications:**

*There are clear and informative communications about upcoming meetings, written work, and activities.*

Strongly agree

Agree

Neither agree nor disagree

Disagree

Strongly disagree

*There are regular opportunities to offer feedback about meetings, project ideas and activities.*

Strongly agree

Agree

Neither agree nor disagree

Disagree

Strongly disagree

*My feedback is gathered, acted on and shared back to the group.*

Strongly agree

Agree

Neither agree nor disagree

Disagree

Strongly disagree

**Governance:**

*Group member’s voices are heard, valued, and respected in decision making.*

Strongly agree

Agree

Neither agree nor disagree

Disagree

Strongly disagree

**Impact:**

*Group members are involved in deciding how the impact of their involvement should be assessed (I.e., Group members are involved in deciding how to evaluate the progress and impact of the group).*

Strongly agree

Agree

Neither agree nor disagree

Disagree

Strongly disagree

*Time and activities such as meetings are allocated to reflect and evaluate the patient and public involvement.*

Strongly agree

Agree

Neither agree nor disagree

Disagree

Strongly disagree

*My involvement has an impact on research.*

Strongly agree

Agree

Neither agree nor disagree

Disagree

Strongly disagree

**Working together:**

*The aims and purpose of the group have been jointly agreed and defined by the group.*

Strongly agree

Agree

Neither agree nor disagree

Disagree

Strongly disagree

*I feel valued and able to share ideas freely without restriction.*

Strongly agree

Agree

Neither agree nor disagree

Disagree

Strongly disagree

*The practical arrangements, preferences, and requirements for working together have been discussed, addressed, and agreed as a group.*

Strongly agree

Agree

Neither agree nor disagree

Disagree

Strongly disagree

*In activities, the team are clear about objectives, how to achieve them and how to present the results.*

Strongly agree

Agree

Neither agree nor disagree

Disagree

Strongly disagree

**Support and learning:**

*There are opportunities to improve one’s skill set to enable more involvement.*

Strongly agree

Agree

Neither agree nor disagree

Disagree

Strongly disagree

**Inclusive opportunities:**

*Patient and public group members are involved in the research from the earliest stages.*

Strongly agree

Agree

Neither agree nor disagree

Disagree

Strongly disagree

*Barriers to involvement such as payment or accessibility have been identified and addressed.*

Strongly agree

Agree

Neither agree nor disagree

Disagree

Strongly disagree

*Information about activities is shared widely in ways that are accessible and appeal to different communities.*

Strongly agree

Agree

Neither agree nor disagree

Disagree

Strongly disagree

*There is choice and flexibility in the opportunities offered.*

Strongly agree

Agree

Neither agree nor disagree

Disagree

Strongly disagree

*The group is a true representation of the Deaf or hard of hearing community.*

Strongly agree

Agree

Neither agree nor disagree

Disagree

Strongly disagree

*If you would like to add comments to clarify your answers on the questions above, please write your comments here.*

……………………………………………………………………….

As we are still in fairly early stages of this group, we are still building the aspects of the **Governance standard**. These are the types of questions we would like to ask in the future. It would be useful to hear your thoughts on this area.

- *Public involvement plans are in place and these plans are regularly monitored, reviewed, and reported on.*
- *Throughout the organisation, there is visible and accountable responsibility for public involvement.*
- *Realistic resources such as money, staff, time are allocated for public involvement.*
- *The privacy of personal information is protected by collecting and using it in a suitable way.*

**(box to add long answer text here)**

**Now is your opportunity to provide some written feedback.**

**What has worked well?**

*Answer:*

**What areas would you like to see improvements in?**

*Answer:*

**What do you see as the impact of being involved within this group?**

You can comment on the impact on you, impact on others with hearing loss, impact on the research community and impact on wider society.

*Answer:*

**Describe any benefits of being part of ALL_EARS.**

*Answer:*

**Now is your opportunity to reflect on your engagement with the group.**

On a scale of 1 to five, how much do you feel you have contributed to the group?

*1 is low contribution, 5 is greatest contribution.*

1 2 3 4 5

**Do you think you are likely to be involved in a year’s time?**
