# Supplementary material for: Patient and public involvement and engagement (PPIE): how valuable and how hard? An evaluation of ALL_EARS@UoS PPIE group, 18 months on
Source: Res Involv Engagem. 2024 Apr 11;10:38. doi: 10.1186/s40900-024-00567-1 (PMC11010367; doi:10.1186/s40900-024-00567-1)
Supplement: Supplementary file 3 — Supplementary Material 3: ALL_EARS@UoS PPIE Action Plan [file 40900_2024_567_MOESM3_ESM.docx]

**ALL_Ears@UoS PPIE Action Plan July 2023 – January 2025**

**UK Standards for Involvement**

**Communications:** use plain language for well-timed and relevant communications, as part of involvement plans and activities.

**Governance:** involve the public in research management, regulation, leadership, and decision making.

**Impact:** seek improvement by identifying and sharing the difference that public involvement makes to research.

**Working together:** work together in a way that values all contributions and builds and sustains mutually respectful and productive relationships.

**Support and learning:** offer and promote support and learning opportunities that build confidence and skills for public involvement in research.

**Inclusive opportunities:** offer public involvement opportunities that are accessible and that reach people and groups according to research needs.

**Communications:** use plain language for well-timed and relevant communications, as part of involvement plans and activities.

Actions:

- Discuss setting up a Facebook group or Facebook page.
- Discuss feedback following meetings – completing feedback form. Then the information is fed back to the group. Feedback cycle.
- Improve communications with the group regarding good and bad funding news. Set up a regular round up email? Every 3 months?

**Governance:** involve the public in research management, regulation, leadership and decision making

Actions:

- Discussion around governance to take place within the PPIE team.
- Ask for comments on the NIHR Governance statements in the evaluation questionnaire.
- What areas for Governance need to be addressed following comments from questionnaire?

**Impact:** Seek improvement by identifying and sharing the **difference** that public involvement makes to research.

Actions:

- What is our assessment of impact? Establish this with involvement of members of the group.
- Set up an evaluation plan – including evaluation steering group and co-designed questionnaire.
- Set up annual evaluation meeting with group.
- Set up annual evaluation meeting with the PPIE team.
- Set up an annual newsletter.
- Set up annual objective and goal setting = action plan.
- Complete formal evaluation for the first 18 months, write up and disseminate.

**Working together:** Work together in a way that values all contributions and builds and sustains mutually respectful and productive relationships.

Actions:

- Write a Terms of Reference – includes practical arrangements for working together, role and responsibilities, ways of working together. Send out to the group to get feedback and agreement on it.
- Review group member preferences regularly (annually?)
- Organise an online meeting to see whether this is something we could and should be doing more often.
- Set up smaller groups to work on specific projects of interest.
- Remind/review roles and responsibilities regularly, particularly when new people join the group. Provide a copy of Terms of reference.

**Support and learning:** offer and promote support and learning opportunities that build confidence and skills for public involvement in research.

Actions:

- Determine training needs of members (Use a slot in a meeting to discuss - What do group members what training in?).
- Plan how we can address these training needs.
- Determine what resources are needed to support learning/development.
- Add section on website for links about PPI (INVOLVE, NIHR)
- Determine ways to regularly reflect on group learning, how is it documented/recorded?
- Set out dedicated time and activities allocated for reflection on learning.
- Share the information following the reflection activities.

**Inclusive opportunities:** offer public involvement opportunities that are **accessible** and that reach people and groups according to research needs.

Actions:

- Hold specific meetings to discuss the groups research ideas and priorities and establish some projects where members are involved from the start.
- Continue to share information about the group widely to ensure opportunities are shared and appeal to different communities.
- Optimise payment process
- Send out transparent information to members about the expenses and payment process.
- Go into the community to reach a diverse group of people to advertise and recruit new members for the group.
- Offer both online and in person meetings.
