# Supplementary material for: Patient and public involvement and engagement (PPIE): how valuable and how hard? An evaluation of ALL_EARS@UoS PPIE group, 18 months on
Source: Res Involv Engagem. 2024 Apr 11;10:38. doi: 10.1186/s40900-024-00567-1 (PMC11010367; doi:10.1186/s40900-024-00567-1)
Supplement: Supplementary file 4 — Supplementary Material 4: Framework for reviewing documents [file 40900_2024_567_MOESM4_ESM.pdf]

Working framework for reviewing research project documents

April 2023

ALL_EARS@UoS

Workflow for researchers to share ideas and receive feedback from PPIE group members about research projects/documents

University of Southampton

Researcher to approach PPIE lead with a project/grant idea to discuss:

- Visiting upcoming PPIE meeting
- Costing in PPIE to grant proposal

→

Researcher to attend PPIE meeting to pitch, discuss and develop your idea with the group.

→

Researcher to prepare research project documents

→

Researcher to send project documents and draft email containing all relevant information to PPIE lead to be emailed out to group members.

Set timeline to get comments back.

→

PPIE lead to email project documents and relevant information to volunteers from the group.

→

Group members send feedback to PPIE lead.

↓

PPIE lead to send comments/ reviewed documents to researcher.

←

Within agreed timeline (either 1 or 2 weeks), the researcher should provide feedback in a short email form to be sent back to members who reviewed the documents to complete the feedback cycle.

Put these short emails in a single word document and send to PPIE lead who will email group members.

AUDITORY IMPLANT SERVICE
USAIS
MAKING CONNECTIONS

What works?

1. Project or idea has been mentioned or discussed at an in-person meeting so group members have a rough idea of what it is about.
2. Email whole group to ask for volunteers to review OR already know who volunteers are.
3. Send out email outlining:
    - Project title
    - Brief summary of project
    - List what documents are attached
    - How the documents should be reviewed i.e. what should members comment on, look out for.
    - The preferred format of the feedback e.g. using a review form, adding comments on word, however the members would prefer to do this.
    - How long members have to send back their feedback (timeline).
    - How they will expect to receive feedback from the researcher.
    - Note to say thank you for giving your time to review and comment on these research documents.

4. PPIE lead to collect written emails in a word document and save reviewed documents and attached. On the final day, the word document outlining emails and comments from group members plus attachments will be emailed to the researcher via SafeSend. Or uploaded to a folder on Teams site if relevant.

5. After 1 or 2 weeks, researcher should send word document with comments/feedback for the group members to the PPIE lead.

6. PPIE lead to email feedback from researcher to group members.

7. At the following meeting, mention how the group members contributed and what the next steps for that research project are.

8. Researcher to keep PPIE lead up to date with progress of project and HOW and WHEN further involvement of the group will occur. PPIE lead to keep group members informed of project progress.

Acceptable timeline for feedback from PPIE members:

- 1 week for 1 or 2 short documents
- 2 weeks for longer documents

What documents and in what form?

- Reviewing form
- Ethics application
- Participant Information Sheet
- Consent form
- Plain English Summary

What documents and in what form?

University of Southampton

- **Peer Review Form** – A form to be completed while you are reviewing the project documents to help make an assessment of the quality of the research proposed.

The review form looks like this:

Medicine

University of Southampton

Peer Review for Applications to the Faculty of Medicine Ethics Committee

Please would you review the enclosed application for the Faculty of Medicine Ethics Committee (FoMEC). It is a requirement that all applications to the committee are independently peer reviewed by 2 people prior to submission.

Peer reviewers are chosen on the basis that they are familiar with the type/area of the research proposed and can make an independent assessment of the quality of the research proposed. Applicants should have provided you with sufficient information to allow you to make your judgement. Please complete the form and qualify your views where necessary, adding comments against individual criteria and outlining overall comments at the end.

To be completed by the Principal Applicant:

Short title of Project	
Supervisor / Principal Investigator	

To be completed by reviewer:

Name of Reviewer	
Area of expertise in relation to the project	
I have/have not* been involved in the design or development of the proposed project<br>*please delete as appropriate	
Reviewer's signature	
Date	

Research Review

Project Title: Evaluation of ALL EARS@UoS Patient and Public Involvement and Engagement Group

Please provide your views on the project proposal, commenting specifically on the areas identified in the left hand column.

A. RESEARCH QUALITY			
**Prompts:**		**Yes / No**	**Specific Comments**
1.	Background & Literature: Is the current state of knowledge outlined, well structured, coherent and well referenced?		
2.	Research Question/Hypothesis: Is there a clear hypothesis/ question/purpose which leads on from the background and literature?		
3.	Objectives: Are the objectives:<br>a) stated clearly?		
	b) appropriate?		
	c) achievable?		
4.	Sample:<br>a) Is the sample population described?		
	b) Is the recruitment process feasible?		
5.	Design:<br>a) is the design stated?		
	b) Is there a rationale for the approach?		
6.	Methodology:<br>a) Are the methods chosen appropriate?		
	b) Is the protocol of procedures clear?		
7.	Research tools: Are the research tools (such as equipment, questionnaires and interviews) well structured, informed, and suitable for analysis?		

AUDITORY IMPLANT SERVICE
USAIS
MAKING CONNECTIONS

AU

What documents and in what form?

University of Southampton

- **Ethics application** – This will contain information on the background, aims, objectives, expected outcomes of the project.

- **Participant Information Sheet** – This will describe the project and what it will involve for the participant involved in the study. This document should be written in clear language that is understandable to a lay audience.

- **Consent form** – This should include clear statements about the participants involvement in the research study.

- **Plain English Summary** – This will be a short description of the research project in non-scientific language that is suitable for a lay audience.

Please note some projects will not include all of the documents listed above.

AUDITORY IMPLANT SERVICE
USAIS
MAKING CONNECTIONS

Timeline for feedback:

University of Southampton

Acceptable timeline for feedback from PPIE members:

- 1 week for 1 or 2 short documents
- 2 weeks for longer documents

Acceptable timeline for feedback from researcher to send back to group members:

- To be agreed with PPIE lead before asking for feedback from PPIE group members.
- Either 1 or 2 weeks.

AUDITORY IMPLANT SERVICE
USAIS
MAKING CONNECTIONS
